# Comparative Effectiveness of Focused Extracorporeal versus Radial Extracorporeal Shockwave Therapy for Knee Osteoarthritis—Randomized Controlled Study

**DOI:** 10.3390/ijerph19159001

**Published:** 2022-07-24

**Authors:** Nai-Yu Ko, Chih-Ning Chang, Chu-Han Cheng, Hui-Kung Yu, Gwo-Chi Hu

**Affiliations:** 1 Department of Rehabilitation Medicine, Mackay Memorial Hospital, Number 92, Section 2, Zhongshan North Road, Zhongshan District, Taipei City 10449, Taiwan; naiyuko@gmail.com (N.-Y.K.); genine0607@gmail.com (C.-N.C.); b101101078@tmu.edu.tw (C.-H.C.); 2 Mackay Junior College of Medicine, Nursing, and Management, No. 92, Shengjing Rd., Beitou Dist., Taipei City 11260, Taiwan; s487@mail.mkc.edu.tw; 3 Department of Medicine, Mackay Medical College, No. 46, Sec. 3, Zhongzheng Rd., Sanzhi Dist., New Taipei City 252, Taiwan

**Keywords:** extracorporeal shockwave therapy, knee osteoarthritis, cartilage, randomized controlled study

## Abstract

Both focused extracorporeal shockwave (f-ESWT) and radial extracorporeal shockwave therapy (r-ESWT) can alleviate symptoms in patients with knee osteoarthritis, but no trials have directly compared f-ESWT with r-ESWT for knee osteoarthritis. This study aimed to compare the effectiveness of f-ESWT and r-ESWT on knee osteoarthritis. Forty-two patients with bilateral knee osteoarthritis were randomly assigned to receive three sessions of either f-ESWT or r-ESWT at 1-week intervals. The patients were evaluated at baseline and at 4 and 8 weeks after the final treatment. The primary outcome was the change in pain intensity, as measured on the visual analog scale (VAS). Secondary outcomes included the Western Ontario and McMaster Universities Osteoarthritis Index (WOMAC), range of motion of the knee joint, and the 6-minute walk test. At the end of 4 weeks, the VAS score was substantially reduced in both groups (f-ESWT, −4.5 ± 2.5 points; r-ESWT, −2.6 ± 2.0 points), with a greater reduction in the f-ESWT group. Both groups showed significant improvement in secondary outcomes; however, the f-ESWT group yielded greater improvement in the VAS score, WOMAC score, and 6-minute walk test. Our results showed that f-ESWT was more effective than r-ESWT in improving pain and physical function in patients with knee osteoarthritis.

## 1. Introduction

Knee osteoarthritis (OA), which is the most common form of arthritis, is characterized by inflammation and major structural changes in the knee joint. Common symptoms such as pain, joint stiffness, and decreased range of motion (ROM) interfere with the individual’s ability to perform daily activities, thereby affecting the quality of life [1]. The prevalence of knee OA increases with age. Approximately one-third of the people of age > 60 years are affected by symptomatic knee OA worldwide [2,3,4]. Additionally, the increasing prevalence of obesity among the aging population is anticipated to increase the demand for health services to treat knee OA [5].

Although knee OA is a major public health problem and a leading cause of long-term pain and disability, there has been no well-established treatment to prevent the onset or progression of the disease to date. According to the Osteoarthritis Research Society International (OARSI) guidelines, the initial management of knee OA is conservative and consists of a combination of both non-pharmacological and pharmacological treatment approaches [6]. Non-pharmacological treatment involves exercise programs (strengthening and low-impact aerobic exercises), self-management (weight reduction and adaptation of activities), and biomechanical interventions (knee braces and foot orthoses) [7,8,9], while pharmacological treatment includes painkillers (analgesics such as acetaminophen and non-steroidal anti-inflammatory drugs) [10,11]. Patients who do not respond to the above treatments are considered for surgical intervention. However, most of these treatments have their limitations in terms of tolerability and durability. Therefore, innovative and effective treatments that can relieve pain and improve the functional status of knee OA patients are urgently needed.

Extracorporeal shockwave therapy (ESWT) is a noninvasive therapeutic modality that uses a sequence of high-energy acoustic waves to induce biological and physiological effects within the tissues and cells of the target area for treatment [12]. ESWT in recent years has emerged as a valuable treatment method for various neurological and musculoskeletal disorders [13,14]. Animal studies reported that ESWT produced chondroprotective [15], anti-inflammatory [16], neovascularization [17], anti-apoptotic [18], and tissue regeneration effects [19], which could be relevant in the treatment of OA. A recent systematic review and meta-analysis of 14 studies that included 782 participants with knee OA suggested that ESWT is an effective treatment for relieving pain and enhancing the functional status of knee OA patients without any significant adverse effects [20]. In general, there are two different types of ESWT: focused extracorporeal shockwave therapy (f-ESWT) and radial extracorporeal shockwave therapy (r-ESWT), which differ in physical characteristics and wave propagation patterns. The pressure wave of f-ESWT generally exhibits a sharp rise in pressure over a very short period. The f-ESWT beam has a concentrated shape in which the pressure converges to an adjustable focus at a selected depth in body tissues. Unlike f-ESWT, r-ESWT has a different linear pressure, relatively low velocity of propagation, and long rise time duration. The maximal pressure of r-ESWT is at the skin surface and then diverges as it penetrates deeper. These differences may lead to different therapeutic effects between f-ESWT and r-ESWT [21,22]. Although shockwave therapy is not currently approved by the International Society for Medical Shockwave Treatment (ISMST) for treating OA, recent studies suggested that f-ESWT or r-ESWT could be effective at reducing pain and improving the functional status of knee OA patients [21,22,23,24]. To our knowledge, no prior studies have directly compared the effects of f-ESWT and r-ESWT in knee OA patients. This led to the genesis of the current study, which involved a randomized clinical trial to compare the effectiveness of f-ESWT to that of r-ESWT in knee OA patients based upon the following parameters: pain, physical function, ROM, and walking speed.

## 2. Materials and Methods

Based on the criteria set by the American College of Rheumatology [25], patients diagnosed with bilateral knee OA were recruited from the rehabilitation department of a tertiary hospital in Taiwan between December 2017 and October 2019. All patients underwent simple radiographs that provided antero-posterior and lateral views of the knee in the standing position. The severity of OA was gauged using the Kellgren–Lawrence grading scale, which is a universally method for classifying radiographic OA [26]. The inclusion criteria were as follows: age > 50 years, symptom duration of at least 6 months, and a Kellgren–Lawrence grade of II to III. The exclusion criteria were as follows: body mass index (BMI) ≥ 35; administration of knee injections in the last 6 months; history of knee injury, surgery, and/or tumors; diagnosis of inflammatory or post-infectious knee arthritis; gouty arthritis; psoriatic arthritis; septic arthritis; inability to walk without a gait aid; and contraindications for ESWT (pregnancy, cancer, coagulation disorders, inflammatory disease, pacemakers, or other electronic implants). Patients with any neurologic, musculoskeletal, or other diseases that affected lower extremity balance, strength, or movement ability were also excluded. The clinical examination and radiographic imaging were evaluated by the same physiatrist. Patients were given thorough explanations about the characteristics and purpose of the study, and thereafter gave their informed consent to participate in the study. The study protocol was reviewed and approved by the relevant ethics committees and was registered in ClinicalTrials.gov (NCT03921749).

After completing the baseline screening and evaluation, eligible patients were randomly assigned to either of the two groups: f-ESWT or r-ESWT. The allocation was guided by a computer-generated random allocation schedule, which was developed by an investigator. The final randomization order was concealed in sequentially numbered envelopes. As each patient entered the trial, the next envelope in the sequence was opened, indicating the treatment to be received by the patient. During the trial, treatment allocation was concealed from the subjects and the outcome assessors. An experienced physiatrist who was not involved in the baseline evaluation and further follow-up assessments performed the ESWT. Shockwave treatments were applied with the Duolith SD1 device (Storz Medical, Tagerwilen, Switzerland). The device could deliver both electromagnetically generated focused or pneumatically driven radial extracorporeal shockwaves. All the patients received three sessions of shockwave treatment at weekly intervals. For the treatment, patients remained in a supine position with the knee joints flexed at 90°. The shockwave probe was administered directly on the most tender areas of the medial tibial plateau and the patellofemoral border of the knee joint. Both knees received the same treatment. During each session, 2000 pulses (1000 shocks in the medial tibial plateau and 1000 shocks in the patellofemoral border) were delivered at 5 Hz. The intensities that were used during f-ESWT (0.10 mJ/mm^2^) and r-ESWT (3.0 Bar) were comparable because when air pressure generated by r-ESWT can be converted to energy flux density using the following equation:

$$J=\frac{1}{Z}\int_{a}^{b}P{\left(t\right)}^{2}dt$$

*Z*: acoustic impedance;

*P*(*t*): the pressure as a function of time;

*a*: the first positive extreme of the first measured pressure peak;

*b*: the second positive extreme of the first measured pressure peak.

In this equation, the energy flux density was obtained by calculating the energy per area. The energy was determined via integration from the pressure curve plotted against time [27]. During the study period, the patients received no additional treatment, such as physical therapy, steroid injection, anti-inflammatory drugs, and exercise specifically for the knee joint.

Clinical assessments were performed at baseline, that is, before treatment, and at the end of 4 and 8 weeks after treatment. An experienced therapist, who was blinded during the treatment allocation, performed the assessments. The primary outcome was the change in pain intensity between baseline and 4 weeks after treatment, as measured on the visual analog scale (VAS). The VAS instrument measures pain using a scale of 0–10 cm; a score of 0 cm indicates no pain, whereas 10 cm is indicative of very severe pain [28]. The patients used the scale to indicate knee pain level after 5 minutes of a weight-bearing position (either standing or walking).

Secondary outcomes included the Western Ontario and McMaster Universities Osteoarthritis Index (WOMAC), ROM of the knee joint, and distance covered in the 6-minute walk test. The WOMAC, which is a validated disease-specific self-administered scale, was designed to evaluate knee OA symptoms. The scale includes 24 items divided into 3 dimensions: pain (5 items), stiffness (2 items), and physical function (17 items). Each item is scored from 0 (none) to 4 (extreme). The total score is calculated by adding the points for all three dimensions [29]. A higher score indicates worse disease status. Knee ROM was measured in the supine position using a standard long-arm goniometer. For active knee flexion, the participant was instructed to simultaneously flex the knee and hip joints as far as possible while keeping the other foot on the supporting surface. A fully extended knee was defined as the zero position. Knee ROM was the angle between the maximum flexion and maximum extension. The ROM was measured three times for the more severe side of the knee. The average of the three measurements was used for the data analysis. The 6- minute walk test measured the maximum distance that the patients could cover by walking for 6 minutes. Herein, the patients were instructed to walk up and down a 25 meters path for 6 minutes. The 6-minute walk test is a reliable measurement of the functional exercise capacity in patients with knee OA [30]. A report completed by the patients at each follow-up session helped to assess the adverse effects of ESWT.

Sample-size calculations were based on detecting a mean difference of 2 cm minimal clinically important difference (MCID) on a 10 cm VAS between the two groups with a standard deviation (SD) of 2 cm, which was adjusted based on the results of previous studies [22,31]. Additional parameters used for the sample calculation were a two-tailed test, an alpha level of 0.05, a desired power of 80%, and a dropout rate of 20%. Based on these calculations, the minimum sample size was estimated to be 21 patients per group.

All analyses used data obtained from the randomly assigned patients under the intention-to-treat assumption. The Kolmogorov–Smirnov test revealed a normal distribution of the variables (*p* > 0.05). The continuous variables were expressed as mean ± SD and/or 95% confidence intervals (CI), while the categorical variables were expressed as proportions. Baseline demographic and clinical variables were compared between the two treatment groups using the independent *t*-test or Mann–Whitney U test for continuous data and the chi-square test for categorical data.

Linear mixed models incorporating all the values recorded for each patient at baseline and the end of 4 and 8 weeks of treatment were utilized to assess the differences in the effect of f-ESWT and r-ESWT on the treatment of knee OA over time. The mixed model included the group (f-ESWT vs. r-ESWT) and time (three time points of measurement) as fixed effects and subjects as random effects, with the baseline value as a covariate. The difference in the treatment effect over time between the two groups was studied using the product of the interaction term group and time. Planned contrast tests were used to compare the differences in outcome variables within and between the groups at each time point. In addition, Cohen’s d effect size was estimated by using the two-by-two between-group magnitude of change from baseline to each time end-point divided by the pooled standard deviation, and interpreting values of <0.2 as small, 0.5 as moderate, and >0.8 as large effect sizes.

The chi-square test or Fisher’s exact test was used to assess and compare the adverse effects of f-ESWT and r-ESWT reported between the groups. All statistical analyses were performed using SAS version 9.2 (SAS, Cary, NC, USA). Values with *p* < 0.05 were considered statistically significant.

## 3. Results

**Figure 1:**
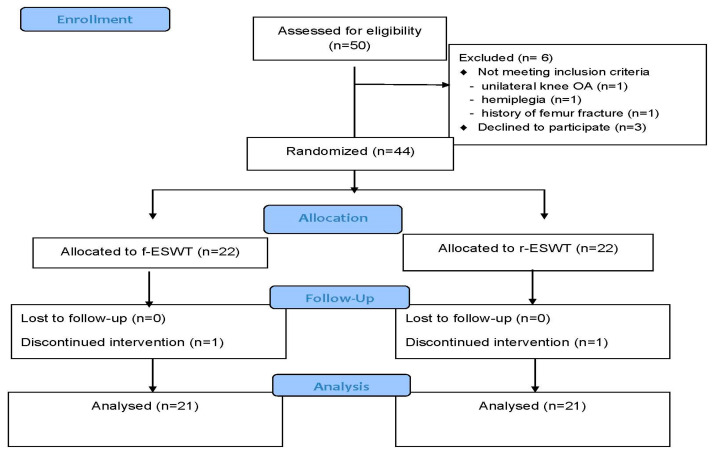
Flow diagram of patients included in the study.

Figure 1 shows the study flowchart. Out of the 50 patients screened, 44 patients were randomly assigned to the f-ESWT and r-ESWT groups. Each group had 22 patients. However, before initiating the intervention, one patient from each group decided to withdraw from the study. Therefore, 42 patients (21 in each group) completed the study and were included in the final analysis.

Table 1 summarizes the demographic and clinical characteristics of the remaining patients. No significant difference was found between the participants of the f-ESWT and r-ESWT groups with respect to age, sex, weight, height, BMI, and average duration of knee OA.

Table 2 presents the primary and secondary outcome variables by point of measurement for both groups. The change of VAS score from baseline to 4 weeks post-treatment was −4.5 ± 2.5 points in the f-ESWT group and −2.6 ± 2.0 points in the r-ESWT group, with a greater reduction in the f-ESWT group. There were no significant main effects for group for the primary outcome of VAS score and the secondary outcome of WOMAC score, the distance of the 6-minute walk test, and the ROM of the knee joint, indicating that there were no significant differences in any of the outcome variables between the two groups at baseline evaluation. There were significant main effects for time for all outcome variables, indicating there were significant changes in all outcome variables over time during the study period. In addition, the linear mixed model analysis revealed a statistically significant group-by-time interaction for VAS score (*p* = 0.01), WOMAC score (*p* < 0.001), and the distance in the 6-minute walk test (*p* = 0.003), indicating participants of the f-ESWT group showed greater improvement in all three parameters compared with the r-ESWT group. However, the group-by-time interaction was not significant for the ROM of the knee joint (*p* = 0.46), indicating that the patients of both groups experienced similar increases in the ROM of the knee joint during the study period.

Compared with the baseline, participants of both f-ESWT and r-ESWT groups reported significant improvements in the VAS score, WOMAC score, ROM of the knee joint, and the distance in the 6-minute walk test at the end of 4 and 8 weeks of treatment. However, the results for f-ESWT were superior to the r-ESWT in terms of VAS score, WOMAC score, and the distance in the 6-minute walk test (Table 3). No adverse events were reported during the study.

## 4. Discussion

The results of this study indicated that both f-ESWT and r-ESWT played significant roles in reducing the knee pain level and improving the WOMAC score, ROM of the knee joint, and the 6-minute walk test distance in patients with knee OA. However, f-ESWT was superior to r-ESWT with respect to pain reduction and an improved WOMAC score and 6-minute walk test distance. Both treatment groups showed similar improvement in knee ROM. 

Knee OA is a common and disabling disease in middle-aged and older adults. A decrease in pain intensity and improvement in physical function are the two main treatment goals for knee OA [10]. Several studies described the positive effects of ESWT on knee OA. Wang et al. [32] conducted a systematic review and meta-analysis of nine trials in 431 patients and they found that ESWT could significantly improve pain (mean difference: −2.78, 95% CI: −3.93 to −1.64) and the Western Ontario and McMaster Universities Osteoarthritis Index function outcome (mean difference: −12.02, 95% CI −31.29 to −7.24), which was similar to the results of our study. The exact mechanisms triggered by ESWT are not fully understood, but several studies demonstrated the effects of ESWT on OA pathogenesis and pain reduction. Animal studies reported that ESWT inhibits the production of tumor necrosis factor alpha and nitric oxide in the knee synovial cavity [33]. Nitrous oxide and tumor necrosis factor alpha mediate the inflammatory response and is associated with chondrocyte apoptosis. Therefore, the decreased levels of tumor necrosis factor alpha and nitrous oxide may partially explain the chondroprotective effects of ESWT [34]. In addition, ESWT can promote neovascularization and the secretion of growth factors, which have great potential in improving chondrocyte viability and cartilage repair mechanisms [35]. Moreover, ESWT causes a reduction in substance P in the target tissue in conjugation with the suppression of calcitonin gene-related peptide release in the dorsal root ganglia [36]. Substance P and calcitonin gene-related peptides are bioactive substances that are thought to play important roles in chronic inflammation; the depletion of substance P was shown to reduce experimentally induced inflammation in laboratory animals [36]. This may help to explain the relief of clinical pain experienced after the application of ESWT.

Although several studies demonstrated that ESWT is a safe and effective treatment for knee OA, further research is necessary, especially when different shockwave types are used. In general, ESWT can be classified into two groups: f-ESWT and r-ESWT, which differ in terms of physical properties and acoustic wave propagation patterns [37]. Zhao et al. performed a randomized control trial to compare r-ESWT with a placebo in 70 patients with knee OA. In this trial, patients treated with r-ESWT reported significant improvements in the average pain score, WOMAC score, and the Lequesne index compared to the placebo group at a 3-month follow-up [21]. Zhong et al. reported a similar outcome: patients treated with 2000 shocks of r-ESWT had a better VAS score, WOMAC score, and Lequesne index than the placebo group [22]. Uysal et al. compared the efficacy of r-ESWT treatment with sham-ESWT for knee OA. They found r-ESWT to be more effective than sham-ESWT at improving pain, walking speed, physical function, and isokinetic muscle strength [23]. Investigating the effectiveness of f-ESWT in patients with knee OA, Lee et al. found that f-ESWT in combination with physical therapy was superior to physical therapy alone in reducing pain and improving the functional status of knee OA patients [38]. Cho et al. found that f-ESWT in chronic stroke patients with knee OA reduces pain, improves function, and increases vascular activity at the target site [39]. Lee et al. compared f-ESWT with intra-articular injections of hyaluronic acid for knee OA. Both treatments yielded similar improvements in the VAS, WOMAC, Lequesne index, and 40-meter walk test distance scores [40]. Our results are consistent with the findings of previous research studies, and thus, suggest that both f-ESWT and r-ESWT are effective at alleviating pain and improving the physical functions and knee ROM in knee OA patients. This study exclusively reported that f-ESWT yielded a greater improvement in the scores of VAS, WOMAC, and 6-minute walk test distance compared with r-ESWT. However, the results contradict the findings of Liao et al. [41], where r-ESWT exerted superior effects on the WOMAC score than f-ESWT. The efficacy of ESWT treatment is influenced by parameters such as frequency, intensity, and total shocks applied. However, these parameters were heterogeneous among the included trials. When heterogeneity exists in the parameters, the outcome of the study varies. A rudimentary pooling strategy may result in misleading conclusions regarding the effects of r-ESWT or f-ESWT on knee OA. We found that under the same frequency, intensity, and number of total shocks, f-ESWT had a greater effect on pain relief and physical function improvement than r-ESWT. The difference in the propagation of shockwaves, the energy distribution, and the different physical properties of f-ESWT and r-ESWT could possibly explain the current observations. While f-ESWT is generated by electromagnetic, electrohydraulic, or piezoelectric methods and has a concentrated beam shape, r-ESWT is generated via a pneumatic method with a dispersed beam shape. Because of the differences in the wave propagation pattern, f-ESWT has a greater reach and is capable of focusing the energy much deeper into the target area than r-ESWT [42]. Therefore, the focusing mechanism can direct each shock into the bone-cartilage interface, which is the target in knee OA treatment, without any loss of energy. In addition, r-ESWT lacks the characteristic features of shockwaves, such as a short rise time, high peak pressure, and non-linearity, thus reducing its effect on knee OA [37].

Previous studies showed that either f-ESWT or r-ESWT could increase ROM in patients with knee OA. The restricted ROM of the knee joint is an important determinant of disability in patients with OA. Our study reported that f-ESWT and r-ESWT had similar effects on the ROM in patients with knee OA. This might be because the ROM was limited not only by bony and cartilage non-congruity but also by a variety of contracted structures, including the periarticular muscles, ligaments, and tendons [43]. Previous studies found that r-ESWT has a greater effect on the superficial muscles and tendons [44]. Further studies are warranted to verify the effects of f-ESWT and r-ESWT on knee ROM in OA patients. In line with the previous trials that reported no significant adverse events for ESWT, the current data also suggests that ESWT is safe for knee OA patients.

This study has some limitations that need to be addressed. First, our study design lacked a true control group with either placebo or no treatment. However, the results of previous clinical trials showed that either f-ESWT or r-ESWT had an advantage over a placebo in terms of pain relief and knee function improvement in patients with knee OA. Second, even though we were able to blind the assessor, we were not able to blind the patients to their assigned treatment. To decrease the bias, we included the ROM and 6-minute walk test as objective secondary outcomes. Third, the optimal treatment protocol has not been established, and the parameter settings of ESWT applied in this trial were chosen based on the previous relevant studies. We do not know whether different treatment sessions, intensities, or frequencies would have revealed differences between the two groups. Fourth, our study lasted only 3 months, and sustained effects for a longer duration remain unknown. Moreover, we were not able to closely monitor the levels of physical activity in these outpatients after they went home. Finally, the generalizability of the study may be limited by the data from a single institution.

Despite these limitations, our study is the first to directly compare the effects of f-ESWT and r-ESWT with identical treatment parameters in patients with knee OA. The current results add to the growing evidence that f-ESWT and r-ESWT are effective and safe modalities for treating knee OA. Our study provided further evidence that shockwave types might be a significant influencing factor while treating knee OA patients with ESWT.

## 5. Conclusions

Both f-ESWT and r-ESWT are effective at alleviating pain and improving physical functions, knee ROM, and the 6-minute walk test distance in patients with knee OA. The f-ESWT yielded a greater reduction in pain and improvements in physical function and the 6-minute walk test distance compared with r-ESWT. Future studies will need to compare the long-term effects of f-ESWT and r-ESWT and investigate the exact mechanisms in regards to knee OA.

## Figures and Tables

**Table 1:** Baseline demographic and clinical characteristics of participants by treatment group.

Variables	f-ESWT Group	r-ESWT Group	*p*-Value
	(n, 21 Patients)	(n, 21 Patients)	
Age (years)	64.1 ± 11.4	63.1 ± 11.2	0.78
Weight (kg)	69.5 ± 11.5	68.6 ± 15.7	0.84
Height (meters)	1.61 ± 0.07	1.58 ± 0.07	0.11
BMI (kg/m^2^)	26.6 ± 4.2	27.2 ± 5.1	0.65
Time since knee osteoarthritis diagnosis (years)	5.3 ± 2.3	5.0 ± 4.2	0.83
Gender (%)			0.75
Female	12 (57%)	13 (62%)	
Male	9 (43%)	8 (38%)	
Kellgren–Lawrence grade	(n, 42 knees)	(n, 42 knees)	0.65
Grade II	14 (33%)	16 (38%)	
Grade III	28 (67%)	26 (62%)	

Continuous data are presented as mean ± standard deviations and categorical data as number (%). Abbreviations: f-ESWT, focused extracorporeal shockwave therapy; r-ESWT, radial extracorporeal shockwave therapy; BMI, body mass index.

**Table 2:** Outcome variables at baseline and each follow-up assessment for both groups.

Variables	Time Point	f-ESWT Group	r-ESWT Group	*p*-Value *		
		Mean ± SD	Mean ± SD	Time Effect	Group Effect	Group-by-Time Interaction Effect
VAS score	Baseline	6.3 ± 1.8	5.9 ± 1.6	<0.001	0.19	0.01
	4-week follow-up	1.8 ± 1.5	3.3 ± 2.6			
	8-week follow-up	2.4 ± 2.4	3.3 ± 2.0			
WOMAC score	Baseline	37.5 ± 14.7	33.6 ± 12.7	<0.001	0.09	<0.001
	4-week follow-up	13.9 ± 6.3	24.4 ± 9.2			
	8-week follow-up	12.2 ± 9.4	21.7 ± 9.8			
Range of motion (degrees)	Baseline	114.1 ± 12.3	115.6 ± 12.2	<0.001	0.61	0.46
	4-week follow-up	121.2 ± 10.2	124.6 ± 10.6			
	8-week follow-up	126.4 ± 10.9	125.9 ± 12.5			
Six-minute walk test (meters)	Baseline	403.1 ± 117.6	416.9 ± 107.9	<0.001	0.44	0.003
	4-week follow-up	490.4 ± 94.6	448.6 ± 113.0			
	8-week follow-up	491.7 ± 97.2	448.1 ± 106.9			

Abbreviations: f-ESWT, focused extracorporeal shockwave therapy; r-ESWT, radial extracorporeal shockwave therapy; SD, standard deviation; VAS, visual analog scale; WOMAC, Western Ontario and McMaster Universities Osteoarthritis Index. * *p*-value was obtained using the linear mixed model.

**Table 3:** Outcome changes from baseline to each follow-up assessment for both groups.

	Time Interval	Mean Change from Baseline		Between-Group Difference	Effect Size (Cohen’s d)
Variables		f-ESWT Group (Mean, 95% CI)	r-ESWT Group (Mean, 95% CI)	f-ESWT versus r-ESWT	
VAS score	Week 4–baseline	−4.5 (−5.6, −3.4)	−2.6 (−3.5, −1.7)	−1.9 (−3.3, −0.4)	0.67
	Week 8–baseline	−3.9 (−5.0, −2.7)	−2.6 (−3.5, −1.7)	−1.3 (−2.6, −0.1)	0.55
WOMAC score	Week 4–baseline	−23.5 (−30.3, −16.8)	−9.1 (−13.6, −4.6)	−14.4 (−22.2, −6.5)	0.72
	Week 8–baseline	−25.3 (−32.7, −17.8)	−11.9 (−16.6, −7.1)	−13.4 (−22.0, −4.9)	0.72
Range of motion (degrees)	Week 4–baseline	7.1 (2.1, 12.0)	9.0 (5.0, 12.9)	−1.9 (−8.0, 4.3)	0.26
	Week 8–baseline	12.2 (6.7, 17.2)	10.3 (5.9, 15.2)	2.0 (−4.7, 8.8)	0.09
6-minute walk test (meters)	Week 4–baseline	87.2 (46.8, 132.9)	31.6 (14.0, 49.9)	55.6 (12.8, 98.4)	0.78
	Week 8–baseline	88.6 (44.4, 132.8)	31.2 (14.3, 48.1)	57.4 (11.5, 103.3)	0.72

Abbreviations: f-ESWT, focused extracorporeal shockwave therapy; r-ESWT, radial extracorporeal shockwave therapy; CI, confidence interval; VAS, visual analog scale; WOMAC, Western Ontario and McMaster Universities Osteoarthritis Index.

## Data Availability

The datasets that were generated and analyzed during the current study are not publicly available, but are available from the corresponding author on reasonable request.
